# ER Stress, the Unfolded Protein Response and Osteoclastogenesis: A Review

**DOI:** 10.3390/biom13071050

**Published:** 2023-06-28

**Authors:** Wangli Huang, Yining Gong, Liang Yan

**Affiliations:** Department of Spine Surgery, Honghui Hospital, Xi’an Jiaotong University, Xi’an 710054, China

**Keywords:** ER stress, UPR, osteoclastogenesis, bone resorption

## Abstract

Endoplasmic reticulum (ER) stress and its adaptive mechanism, the unfolded protein response (UPR), are triggered by the accumulation of unfolded and misfolded proteins. During osteoclastogenesis, a large number of active proteins are synthesized. When an imbalance in the protein folding process occurs, it causes osteoclasts to trigger the UPR. This close association has led to the role of the UPR in osteoclastogenesis being increasingly explored. In recent years, several studies have reported the role of ER stress and UPR in osteoclastogenesis and bone resorption. Here, we reviewed the relevant literature and discussed the UPR signaling cascade response, osteoclastogenesis-related signaling pathways, and the role of UPR in osteoclastogenesis and bone resorption in detail. It was found that the UPR signal (PERK, CHOP, and IRE1-XBP1) promoted the expression of the receptor activator of the nuclear factor-kappa B ligand (RANKL) in osteoblasts and indirectly enhanced osteoclastogenesis. IRE1 promoted osteoclastogenesis via promoting NF-κB, MAPK signaling, or the release of pro-inflammatory factors (IL-6, IL-1β, and TNFα). CREBH promoted osteoclast differentiation by promoting NFATc1 expression. The PERK signaling pathway also promoted osteoclastogenesis through NF-κB and MAPK signaling pathways, autophagy, and RANKL secretion from osteoblasts. However, salubrinal (an inhibitor of eIF2α dephosphorylation that upregulated p-eIF2α expression) directly inhibited osteoclastogenesis by suppressing NFATc1 expression and indirectly promoted osteoclastogenesis by promoting RANKL secretion from osteoblasts. Therefore, the specific effects and mechanisms of p-PERK and its downstream signaling on osteoclastogenesis still need further experiments to confirm. In addition, the exact role of ATF6 and BiP in osteoclastogenesis also required further exploration. In conclusion, our detailed and systematic review provides some references for the next step to fully elucidate the relationship between UPR and osteoclastogenesis, intending to provide new insights for the treatment of diseases caused by osteoclast over-differentiation, such as osteoporosis.

## 1. Introduction

The endoplasmic reticulum (ER) is responsible for the synthesis, folding, and modification of secretory and transmembrane proteins through various chaperone proteins and enzymes in ER organelles. Its function is extremely precisely regulated, but a variety of internal factors and changes in the external environment will destroy the protein folding ability of the organelles and trigger ER stress, which is characterized by the accumulation of misfolded or unfolded proteins [1,2]. Notably, cells constantly monitor the number of misfolded proteins in the ER to ensure that the protein folding capacity is in balance with the demand. When the accumulation of misfolded proteins in the ER exceeds a critical threshold, a signaling pathway called the unfolded protein response (UPR) will be initiated [3]. Adaptive UPR maintains protein homeostasis, relieves ER stress, and contributes to cell survival by improving protein folding, reducing protein overload, and increasing the degradation of misfolded proteins through the reprogramming of gene expression and inhibiting protein translation [4]. The basic UPR signaling cascade in mammals is initiated by the following three ER transmembrane protein sensors: inositol-requiring protein 1α (IRE1α), activating transcription factor 6 (ATF6), and the protein kinase RNA-like ER kinase (PERK) [5].

Bone is a dynamic, metabolically active, and functionally diverse organ that is mainly composed of extracellular matrix proteins and calcium phosphate. The bone remodeling process, mainly consisting of bone formation by osteoblasts and bone resorption by osteoclasts, maintains the dynamic balance of the bone mineral density and minerals [6]. It is worth noting that the survival, proliferation, differentiation, and bone resorption activity of osteoclasts are mainly regulated by the macrophage colony-stimulating factor (M-CSF)–colony-stimulating factor 1 receptor (CSF1R), with the receptor activator of nuclear factor-kappa B ligand (RANKL)–receptor activator of nuclear factor-kappa B (RANK). The binding of RANKL to RANK will initiate downstream cascade signaling pathways, such as nuclear factor-kappa B (NF-κB), mitogen-activated protein kinase (MAPK), reactive oxygen species (ROS), the Ca^2+^-nuclear factor of activated T-cells cytoplasmic 1 (NFATc1), and Src.

During osteoclastogenesis, large amounts of active proteins are synthesized and secreted to promote osteoclast differentiation and bone resorption functions, which may activate ER stress and UPR. Interestingly, in addition to being proven to be an adaptive mechanism to alleviate ER stress and promote cell survival, UPR is also involved in osteoclast differentiation and bone resorption. In recent years, several studies on ER stress, UPR, and osteoclasts have been conducted, and scholars have found that ER stress plays an extremely important role in the mechanism of osteoclastogenesis. Therefore, we herein present a detailed review and discussion of ER stress-related mechanisms, signaling pathways of osteoclast differentiation, and the role of branches (IRE1α, PERK, and ATF6) of UPR signaling in osteoclastogenesis.

## 2. ER Stress and UPR

As one of the largest organelles in eukaryotic cells, the ER consists of reticular and tubular structures that span the cell and collaborate with other organelles to maintain normal physiological functions. ER has two main domains: the smooth ER, without ribosome attachment, is involved in lipid synthesis, metabolism, and calcium storage; the rough ER, with ribosome attachment, is responsible for the synthesis and transport of secretory proteins, integral membrane proteins, and cytoplasmic protein. At least a third of all proteins, including calcium-handling proteins, the transmembrane receptors, growth factors, and hormones, are synthesized, modified, folded, and matured by the ER, and then transported to various membrane compartments or secreted [7,8,9,10].

The ER is a complex organelle with many different functions and must be tightly regulated to perform its correct function. The disruption of protein folding processes or calcium homeostasis in the ER leads to the accumulation of unfolded and misfolded proteins, triggering ER stress [11]. Notably, in addition to the inhibition of N-glycosylation and changes in the ER Ca^2+^ concentration, increased rates of protein synthesis, missense polymorphisms in individual proteins, depletion of energy (ATP), altered osmolarity, nutritional deficiencies, oxidative stress, viral infections, and elevated temperatures all lead to the accumulation of unfolded and misfolded proteins in the ER, resulting in ER stress [12,13].

**Figure 1:**
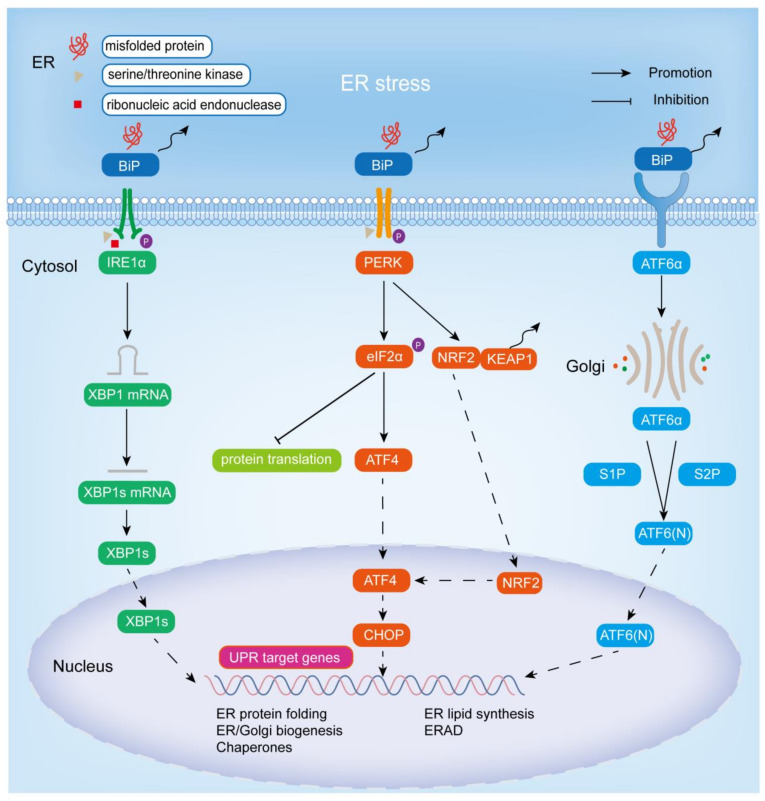
Endoplasmic reticulum stress and the adaptive UPR signaling pathway. Excessive accumulation of unfolded or misfolded proteins in the ER activates the IRE1α-XBP1, PERK-eIF2α, PERK-NRF2, and ATF6 signaling pathways by binding to BiP, thereby promoting the expression of chaperones, the synthesis of ER and Golgi structural components, and the ERAD pathway to improve protein folding and reduce cellular damage from stress.

In response to ER stress, an adaptive mechanism known as the UPR is activated in cells to reduce unfolded or misfolded proteins [14]. In addition to maintaining intracellular protein homeostasis, UPR can also participate in other physiological/pathological responses, such as innate immunity, energy metabolism, and cell differentiation [15]. ER stress activates UPR mainly through three ER stress sensors: IRE1α, ATF6, and PERK. As transmembrane proteins, ER stress transducers have three domains: a luminal domain that senses unfolded and misfolded proteins in the ER lumen, a transmembrane domain, and a cytoplasmic domain, that is responsible for transmitting signals to the cytoplasmic transcription or translation apparatus [16]. Under non-stress conditions, the three ER stress sensors, IRE1α, ATF6, and PERK, bind to the molecular chaperone-binding immunoglobulin (BiP), also known as the 78 kDa glucose regulatory protein (GRP78), in the lumen of the ER, thereby blocking UPR signaling. When ER stress occurs, BiP is isolated by misfolded proteins, allowing the three transmembrane proteins to be activated by homodimerization, autophosphorylation, and cleavage processes, thereby activating the corresponding UPR branches and enhancing protein folding [17]. Below is a brief introduction to three ER stress sensors and their related cascade signaling pathways (Figure 1).

### 2.1. IRE1α Signaling Cascade Pathway

IRE1, encoded by the *ERN1* gene, is the most evolutionarily conserved of the three ER stress sensors and is widely expressed in a variety of species, from yeast to humans [18,19]. There are two types of IRE1: IRE1α and IRE1β. The former is expressed in most tissues and cells, and the other is only expressed in gastrointestinal epithelial cells [19]. As an ER-resident type I transmembrane protein, IRE1α has two enzymatic activities in its cytoplasmic domain, serine/threonine kinase activity and ribonucleic acid endonuclease activity [20]. When unfolded and misfolded proteins accumulate in excess, they bind to BiP, leading to IRE1α dimerization and activation of its protein kinase activity (via autophosphorylation) and ribonucleic acid endonuclease activity [16]. IRE1α then splices an unconventional 26-nucleotide intron from X-box binding protein 1 (XBP1) through its ribonucleic acid endonuclease activity to generate the functionally active protein XBP1 (also known as XBP1s). Subsequently, XBP1s is transferred into the nucleus, thereby promoting the transcription of UPR target genes (including luminal ER protein chaperones (including *BIP*), disulfide isomerases, glycosylases, ER and Golgi structural components, *HERP* (homocysteine-induced ER protein), *DNAJB9* (DnaJ heat shock protein family (Hsp40) member B9)/*ERDJ4*) in the nucleus and the ER-related degradation (ERAD) pathway, thereby promoting ER lipid synthesis, peptide folding, and membrane expansion to reduce ER stress [21,22,23,24]. Recently, XBP1s was found to act on other targets besides the above transcriptional targets: pro-inflammatory cytokines, the HIF-1α hypoxia response pathway, cell differentiation, and the amino hexose biosynthesis pathway [18].

### 2.2. PERK Signaling Cascade Pathway

PERK (encoded by the *EIF2AK3* gene), also referred to as PEK, EIF2AK3, like IRE1α, is an ER-resident type I transmembrane protein, one of the four known eukaryotic initiation factor 2 alpha (eIF2α) protein kinases, and the other three are PKR (protein kinase double-stranded RNA-dependent), GCN2 (general control non-derepressible-2), and HRI (heme-regulated inhibitor) [18,25,26,27]. The N-terminal region of PERK is located in the lumen of the ER and is important for dimerization, regulation, and association with BiP, while the C-terminal region contains a cytoplasmic serine/threonine protein kinase domain [25,28]. When ER stress occurs, binding of BiP to unfolded or misfolded proteins allows the release of PERK and promotes its activation by autophosphorylation and dimerization. Activated PERK directly phosphorylates itself and ubiquitous translation initiation factor eIF2α at Ser51, and inhibits mRNA translation and protein synthesis in the general cell by interfering with 50-cap assembly to reduce the stress load so that the ER has time to fold the accumulated protein [29,30,31]. Phosphorylation of eIF2α leads to a decrease in its activity, resulting in a decrease in mRNA translation, but some mRNAs with short open reading frames in the 5’ untranslated region are preferentially translated when eIF2α is restricted, such as activating transcription factor 4 (ATF4) [30]. In addition, phosphorylation of PERK leads to the separation of NRF2 (nuclear factor-erythroid 2-related factor 2) from KEAP1 (Kelch-like ECH-associated protein 1), allowing translocation of NRF2 into the nucleus, and thus promoting ATF4 expression [32]. ATF4 belongs to the family of basic region-leucine zipper (bZip) proteins, which is widely expressed in human organs. It is activated in response to various stress signals, such as ER stress, hypoxia, amino acid deficiency, and oxidative stress, and acts as a key transcriptional regulator, targeting genes involved in protein folding, autophagy, amino acid metabolism, redox homeostasis, and apoptosis to trigger adaptive cellular responses [33,34,35]. *CHOP* (C/EBP-homologous protein, also known as GADD153) is among them, which is upregulated by ATF4 expression and tends to equalize the unbalanced ER by inducing the expression of many corrective genes, including *XBP1* and chaperone proteins [3].

### 2.3. ATF6 Signaling Cascade Pathway

ATF6 (activating transcription factor 6) includes ATF6α and ATF6β. The most studied is ATF6α, an ER-resident type II transmembrane protein in the inactivated state, characterized by a cytoplasmic N-terminal bZip transcription factor domain and a large ER luminal domain [9,18,30,36,37,38]. Notably, despite the significant homology of ATF6α and ATF6β, the transcriptional effects differ, with ATF6α being a potent transcriptional activator and ATF6β being a poorer transcriptional activator that may inhibit the activation of ATF6α [37]. Unlike IRE1 and PERK, upon suffering ER stress, the luminal domain of ATF6α is directly separated from BiP and subsequently translocated to the Golgi apparatus. In the Golgi, two proteases, S1P and S2P (site-1 and site-2 proteases), sequentially cleave and process ATF6α and remove the luminal domain and the transmembrane anchor [39]. It should be noted that the modification of ATF6α in Golgi entirely depends on S2P, and partly on S1P [40]. The N-terminal cytoplasmic fragment ATF6 (N) released by cleavage of ATF6α is translocated to the nucleus to activate UPR target genes, such as *BIP*, ER degradation-enhancing α-mannosidase-like protein 1 (*EDEM1*), and protein disulphide isomerase-associated 6 (*PDIA6*), thereby promoting ER expansion and protein quality control [37]. Notably, *XBP1* is also a substrate for ATF6α, and ATF6 and XBP1s, the active forms of XBP1, cooperate to promote ER protein folding for quality control in response to ER stress, and thus promote cell survival [41,42].

Furthermore, five other transcription factors in the ER: LUMAN (also known as CREB3), old astrocyte specifically induced substance (OASIS, also known as CREB3L1), BBF2 human homolog on chromosome 7 (BBF2H7, also known as CREB3L2), cyclic AMP-responsive element-binding protein hepatocyte (CREBH, also known as CREB3L3), and CREB4 (also known as CREB3L4), have a similar structure to ATF6 and also contain transcription factors (bZIP) in their cytoplasmic domains. When exposed to ER stress, they are transferred to the Golgi apparatus, cleaved by S1P and S2P, and release active structures to the nucleus, where they exert their effects [27,43].

## 3. The Bone Remodeling and Osteoclastogenesis

Normal adults have 213 bones (excluding seed bones). Bones not only provide structural support for the torso to protect important internal organs and tissues but also act as levers to allow movement and mobility of the torso through muscles. In addition, bones maintain mineral homeostasis and the acid–base balance in the body, provide growth factors and cytokines, and serve as the microenvironment for hematopoiesis in the bone marrow [44].

Although at the macroscopic level the skeleton appears to remain stationary, it is a dynamic and active tissue that is constantly transforming, a process that involves a variety of bone cells, such as osteoblasts, osteoclasts, osteocytes, etc. [45]. Bone tissue is continuously remodeled by the synergistic action of these cells to maintain bone strength and integrity, with osteoblasts being responsible for bone formation, osteoclasts being responsible for bone resorption, and the process of bone metabolism being maintained by both together, called bone remodeling, while osteocytes act as mechanical sensors and coordinators of the bone remodeling process [46,47]. Osteoblasts are differentiated from bone marrow mesenchymal stem cells (MSCs) by bone morphogenic proteins (BMPs) and wingless-related integration site (WNT) pathways under the action of various signal molecules and transcription factors. They mainly secrete bone matrix (composed of type I collagen and non-collagen matrix proteins), calcium, and phosphate ions to replace the bone absorbed by osteoclasts. After completing their mission, they have three possible fates: undergoing apoptosis, becoming bone-lining cells, and becoming osteocytes [48,49]. Osteocytes, which occupy more than 95% of bone cells in the adult skeleton, are the main source of molecules that regulate skeletal homeostasis and the bone remodeling process (by integrating mechanical cues and hormonal signals and coordinating the differentiation and function of osteoblasts and osteoclasts) [50,51]. Following their formation in the bone marrow, mononuclear hematopoietic myeloid lineage cells are attracted to the bloodstream by signaling molecules, including sphingosine-1 phosphate, and then by chemokines to the bone surface or its vicinity, where they differentiate to produce tissue-specific multinucleated bone-resorbing cells, i.e., osteoclasts [52,53].

**Figure 2:**
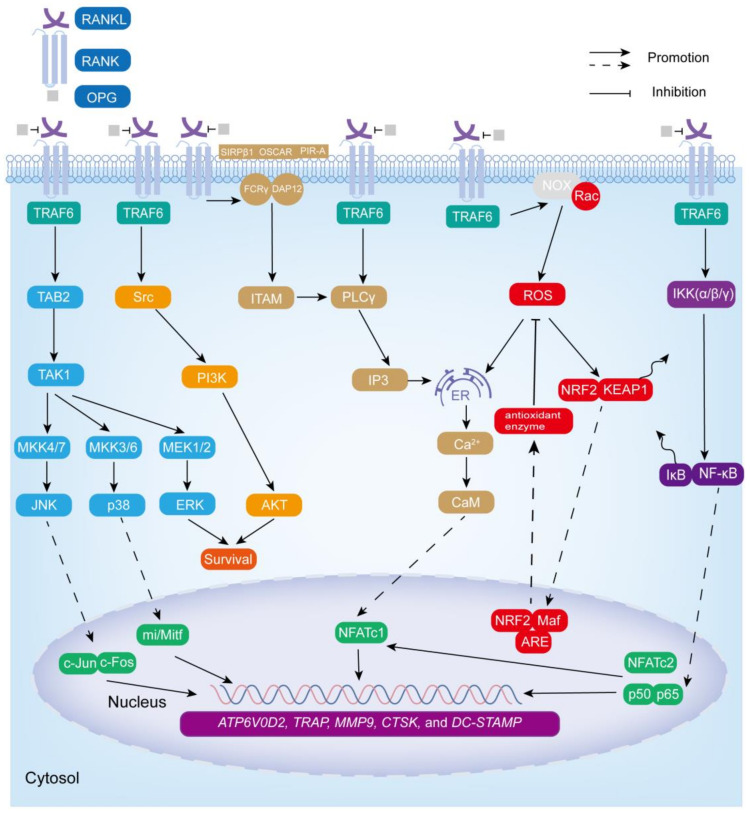
RANKL–RANK-mediated signaling pathway in osteoclastogenesis. RANKL–RANK downstream signaling pathways NF-κB, MAPK, ROS, Ca^2+^-NFATc1, and Src promote osteoclast survival, proliferation, differentiation, and bone resorption activity by inducing the expression of osteoclast-specific genes: *ATP6V0D2*, *TRAP*, *MMP9*, *CTSK*, and *DC-STAMP*.

Remarkably, osteoclastogenesis and the function of osteoclasts are mainly regulated by a family of biologically related tumor necrosis factor (TNF) receptor (TNFR)/TNF-like proteins: RANKL, RANK, and osteoprotegerin (OPG) [53,54]. RANKL, a type II transmembrane protein, also known as osteoprotegerin ligand (OGPL), osteoclast differentiation factor (ODF), and TNF-related activation-induced cytokines (TRANCE), is encoded by the tumor necrosis factor ligand superfamily member 11 (*TNFSF11*) gene on chromosome 13 (13q14.11). RANKL is mainly expressed by osteoblasts, osteocytes, and immune cells. It has an extracellular domain at the carboxyl terminal and is released into the extracellular environment in the form of soluble RANKL after being cleaved by matrix metalloproteinases and other enzymes [55,56]. RANK, a receptor encoded by the *TNFRSF11A* gene on chromosome 18 (18q21.33), is mainly expressed in osteoclast precursors, mature osteoclasts, and immune cells such as DC, macrophages, and microglia. Its carboxyl terminal contains three TNF receptor-associated factor (TRAF) binding domains. OPG, a receptor encoded by the *TNFRSF11B* gene on chromosome 8 (8q24.12), is a soluble decoy receptor without any transmembrane structure, which negatively regulates osteoclastogenesis by binding RANKL [57,58]. In addition to the above-mentioned RANKL, RANK, and OPG, M-CSF plays an essential role in osteoclast formation and bone resorption activity [59]. CSF1R (also known as c-FMS), a receptor tyrosine kinase, is the receptor of M-CSF. The signal transduction mediated by M-CSF-CSF1R is essential for the survival, function, proliferation, and differentiation of bone marrow cells (including osteoclasts), and M-CSF-CSF1R signaling can induce the expression of RANK [60]. Upon secretion, RANKL binds to RANK, which is expressed via M-CSF stimulation on the surface of osteoclast precursor cells [61,62]. The combination of the two leads to the trimerization of RANK, which recruits several adaptor molecules, including TRAF, to specific sites in the RANK cytoplasmic region to initiate downstream cascade signals [58]. The TRAF family consists of seven members (TRAF1, 2, 3, 4, 5, 6, and 7), which mainly play a role in TNF family cytokines and pathogen-associated molecular pattern (PAMP)-induced signals. It is worth noting that the cytoplasmic tail of RANK contains three TRAF6 binding sites and two other TRAF family member (including TRAF2, TRAF3, and TRAF5) binding sites. Although TRAF2, TRAF3, TRAF5, and TRAF6 can activate transcription factors required for osteoclast differentiation, it is generally believed that TRAF6 is the most important in the RANKL-mediated osteoclast differentiation signaling pathway [62,63]. The recruitment of TRAF6 in the RANK cytoplasmic region leads to the activation of multiple signaling pathways, including NF-κB, MAPK, Src, ROS, and Ca^2+^-NFATc1 signaling pathways, resulting in the activation of osteoclast differentiation, survival, proliferation, and bone resorption functions (Figure 2).

### 3.1. NF-κB Signaling Cascade Pathway

The NF-κB family includes NF-κB1 (p50), NF-κB2 (p52), RelA (p65), RelB, and c-Rel [64]. RANKL–RANK promotes the NF-κB signaling pathway through the classical pathway or the alternative pathway in osteoclasts [65,66]. In the classical signaling pathway, RANK recruits TRAF6 to activate the IKK complex (catalytic components (IKKα, IKKβ) and a regulatory component (IKKγ)), thereby phosphorylating IκB (binding to NF-κB in the cytoplasm at rest to inhibit its activity), and then IκB is degraded by the proteasome, allowing p50 and p65 to be rapidly released and translocated to the nucleus, binding to the target gene and mediating osteoclast formation [65,66,67,68].

### 3.2. MAPK Signaling Cascade Pathway

TRAF6 recruitment also induces the formation of complexes containing TGF-β-activated kinase (TAK) 1 and TAK-1-binding protein (TAB) 2 to activate all three MAPK pathways, including JNK (by MKK4/7), ERK (by MEK1/2), and p38 (by MKK3/6) [53,68,69]. Among them, JNK and p38 are related to osteoclastogenesis (activation of downstream transcription factors c-jun/c-fos and transcriptional regulator mi/Mitf, respectively) and ERK is related to the survival, motility, and cytoskeleton rearrangement of osteoclasts [53,70].

### 3.3. ROS Signaling Cascade Pathway

RANKL–RANK binding also induces the production of ROS to stimulate osteoclast differentiation [70,71]. ROS mainly includes highly active free radicals, such as superoxide anions (O2^•−^), and non-free radicals such as hydrogen peroxide (H_2_O_2_) [72]. It is derived from NADPH oxidases (NOX), cytochrome P-450 oxygenase, etc. Notably, NOX is a major source of human ROS, which controls ROS production through the assembly of PHOX subunits (such as p47^phox^) [73,74]. It is worth mentioning that TRAF6 plays a key interlocking role in RANKL-induced ROS production, but TRAF6 itself is not directly involved in ROS production [75]. After RANKL binds to RANK, the NOX complex generates ROS in response to its cytoplasmic component, Ras-related C3 botulinum toxin substrate 1 (Rac1), which then acts on the downstream signaling pathways NF-κB (by activating IKK and phosphorylating IκB), MAPK (by inactivating MAPK phosphatases), and PI3K (by inhibiting phosphatase and tensin homolog) to promote osteoclast formation and survival. ROS also stimulates ER Ca^2+^ release (dependent on inositol 1,4,5 trisphosphate (IP3) receptor regulation) and synergizes with Ca^2+^ to enhance transcription of the key osteoclast transcription factor NFATc1 (also known as NFATc or NFAT2), promoting osteoclast precursor fusion and functional gene expression [70,75,76,77,78]. Under normal conditions, KEAP1 binds to NRF2 on actin fibers in the cytoplasm. After RANKL stimulation, NRF2 is exposed to oxidative stress, resulting in KEAP1 conformational changes and covalent modifications, followed by NRF2 escape from the proteasome and translocation to the nucleus. There, NRF2 heterodimerizes with Maf through its leucine zipper domain, subsequently binding to an antioxidant response element (ARE) to form a trimer that activates antioxidant enzyme genes (such as *NQO1*, *HO-1*, *SOD2*, and *GSH reductase 1*) to diminish the enhancement of osteoclastogenesis by ROS [70,76,79].

### 3.4. Ca^2+^-NFATc1 Signaling Cascade Pathway

In addition, the activation of TRAF6 also stimulates phospholipase Cγ (PLCγ) to produce IP3, which binds to the 1,4,5-trisphosphate receptors (ITPRs) on the ER surface, allowing Ca^2+^ stored in the ER to be released into the cytoplasm, thereby inducing Ca^2+^ oscillations [62]. Ca^2+^ released into the cytoplasm subsequently binds to calmodulin (CaM), leading to the activation of CaM-dependent enzymes, such as the phosphatase calcineurin, which induces NFATc1 translocation into the nucleus and promotes the transcription of osteoclast-specific genes [80]. Furthermore, RANK interacts with immunoglobulin-like receptors OSCAR, signal-regulator protein β1(SIRPβ1), and paired immunoglobulin-like receptor (PIR-A) to activate Fc receptor common γ chain (FcRγ) and DNAX-activating protein 12 (DAP12), followed by the phosphorylation of an immunoreceptor tyrosine-based activation motif (ITAM), which activates syk and PLCγ to produce IP3, that induces the release of Ca^2+^ from the ER and activates NFATc1 [68,71]. Notably, NF-κB and NFATc2 (also known as NFATp or NFAT1) synergistically activate the initial induction of NFATc1 [71,81]. Eventually, NF-κB, c-fos, c-jun, NFATc1, and mi/Mitf promote the expression of osteoclast-specific genes *ATP6V0D2*, *TRAP*, *MMP9*, *CTSK*, and *DC-STAMP*, thereby contributing to osteoclast survival, maturation, and degradation of the bone matrix [82].

### 3.5. Src Signaling Cascade Pathway

After the initiation of RANKL signaling in osteoclasts, Src protein binds to TRAF6 and acts on downstream phosphatidylinositol 3-kinase (PI3K) and the serine/threonine protein kinase AKT to promote the survival of osteoclasts [53,68].

## 4. The Role of UPR in Osteoclastogenesis and Bone Resorption

### 4.1. IRE1α-XBP1

RANKL–RANK-induced calcium oscillations are extremely important for osteoclast formation. Interestingly, calcium oscillations also promoted the activation of ER stress (IRE1α-XBP1) through 1,4,5-trisphosphate receptors 2 and 3 (ITPR2 and ITPR3), allowing XBP1 to bind to the promoter region of *NFATC1*, promoting *NFATC1* transcription and differentiation of osteoclasts in murine models [83]. In the absence of ITPR2 and ITPR3, a lower level of XBP1 was also detected, indicating that calcium channels other than ITPR2 and ITPR3 (such as ITPR1 and ITPR homologous Ryanodine receptor), or other mechanisms such as PERK and ATF6 (XBP1 is also a downstream target of PERK/ATF4 and ATF6), were also involved in RANKL-stimulated ER stress activation [83,84,85].

Thapsigargin (Table 1, where we list the compounds associated with ER stress in this review along with their mechanisms of action and other biological activities), can initiate ER stress and stress-induced apoptosis by inhibiting the sarco/endoplasmic reticulum Ca^2+^-ATPase (SERCA) pump [86]. Furthermore, in a model using osteoclasts differentiated from BMMs (bone marrow-derived macrophages) in vitro, Eun-Gyeong Lee et al. found that thapsigargin-induced ER stress positively regulated NFATc1 and c-fos through the BiP-IRE1α signaling pathway to promote osteoclast differentiation and bone resorption [87]. Another study showed that thapsigargin-induced BiP-IRE1 through the NF-κB pathway, promoted the expression of NAFA1, c-fos, and TRAP, and eventually promoted osteoclastogenesis and bone resorption activity in the bone marrow cells of mice treated with RANKL and M-CSF [88]. A zinc finger protein, MCPIP, played a crucial role in the monocyte chemotactic protein-1 (MCP-1)-induced formation of TRAP-positive osteoclasts derived from human bone marrow monocytes, and mechanistic analysis revealed that MCPIP induced ROS production by p47^phox^, thereby activating ER stress (BiP-IRE1α), which in turn triggered autophagy and stimulated the expression of the osteoclast-specific markers TRAP and CTSK [89]. Titanium components have been widely used in arthroplasty, and aseptic loosening and periprosthetic osteolysis caused by wear particles are among the major complications of arthroplasty. Macrophage apoptosis of the interface membrane, which is closely related to ER stress, has been shown to play an important role in the pathogenesis of aseptic loosening [90]. It was found that titanium particle-induced BiP-IRE1 not only reduced macrophage activity and induced apoptosis but also promoted the transcription of TRAP and RANK to promote osteoclastogenesis and osteolysis [91]. Liang Zhang et al. found that the downregulation of Sirtuin 1 (a nicotinamide adenine dinucleotide (NAD)-dependent deacetylase) caused by wear particles activated ER stress (stress-related molecules BiP, IRE1α, CHOP), thereby activating NF-κB and MAPK and upregulating the expression of NFATc1 and c-fos, and ultimately promoted osteoclast differentiation and bone resorption activity [92].

Notably, the IRE1α signaling pathway also played an essential role in inflammation-mediated bone loss. The inflammatory response at the interface membrane also plays an important role in the loosening of the prosthesis after arthroplasty. ER stress mediated periprosthetic particle-induced inflammatory osteolysis and osteoclastogenesis via BiP-IRE1α and its downstream c-Fos, NF-κB, and Ca^2+^-ROS signaling pathways [103]. Rui Wang et al. found that ER stress (stress-related molecules BiP, IRE1α, CHOP) mediated osteoclast formation and osteolysis induced by wear particles by promoting inflammatory responses (increased expression of pro-inflammatory factors IL-6, IL-1β, and TNF-α) using particle-treated macrophages, periosteal tissue from PIO (particle-induced osteolysis) animal models, and clinical specimens with loose prostheses [104]. Therefore, IRE1 played an active role in the pathogenesis of inflammation-induced bone loss.

In the model of periodontitis induced by oral pseudomonas gingivalis in mice, 4-phenylbutyric acid (4-PBA, an ER stress inhibitor) reversed the significant expression of *BIP*, *XBP1*, total *XBP1*, *CTSK*, and *TRAP* genes and the increase in the osteoclast number and bone resorption in alveolar bone. This suggested that IRE1α-XBP1 played an active role in alveolar bone resorption induced by pseudomonas gingivalis. In addition, 4-PBA also inhibited the formation of multinucleated osteoclasts from M-CSF and RANKL-induced BMMS in a dose-dependent manner via the IRE1α-XBP1 signaling pathway in vitro. Considering that 4-PBA, as a histone deacetylase inhibitor, may affect gene expression related to bone metabolism and the inflammatory response, the use of another chemical chaperone lacking histone deacetylase inhibitory activity, tauroursodeoxycholic acid, also exhibited similar effects to 4-PBA. However, this study failed to elucidate the relationship between ER stress and osteoclast differentiation and pseudomonas gingivalis-induced periodontitis bone resorption at the protein level. As the gene level usually does not necessarily reflect the protein expression level or protein activation status, this study has some limitations [98].

In addition, IRE1α-XBP1 is commonly activated and upregulated in cancers, such as breast cancer, Burkitt’s lymphoma, oral squamous cell cancer, prostate cancer, lung cancer, liver cancer, etc. [105]. It is worth mentioning that ER stress and IRE1α-XBP1 were constitutively activated in multiple myeloma (MM), a malignancy characterized by the production of large amounts of immunoglobulins by malignant plasma cells. MM cell-derived small extracellular vesicles promoted osteoclastogenesis and bone resorption by upregulating the expression of the transcription factor NFATc1 through the initiation of IRE1α-XBP1 [106].

**Figure 3:**
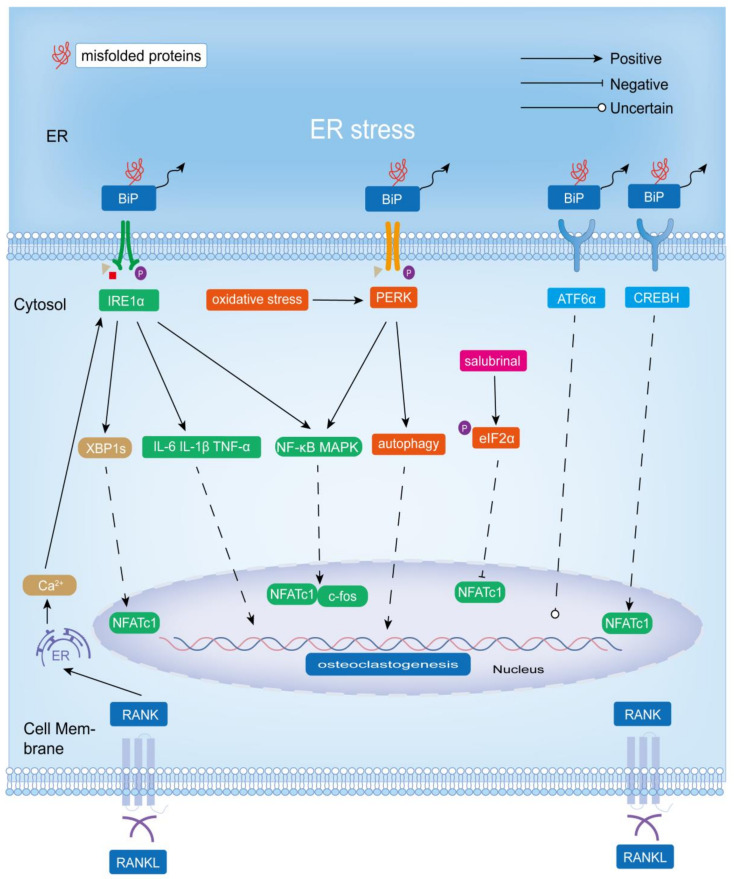
Role of ER stress-induced adaptive UPR signaling mechanisms (IRE1α-XBP1, PERK-eIF2α, and ATF6) in osteoclastogenesis and bone resorption. IRE1α-XBP1 promotes osteoclastogenesis and bone resorption, and BiP-PERK-eIF2α positively affects osteoclastogenesis. However, salubrinal, as an inhibitor of eIF2α dephosphorylation (which upregulates p-eIF2α expression), inhibits osteoblastogenesis by acting directly on osteoclasts. Additionally, the role of ATF6 in osteoclast differentiation and bone matrix degradation is unclear. However, CREBH, which has a similar structure to ATF6, could promote osteoclastogenesis.

In summary, IRE1α-XBP1 played a positive role in osteoclastogenesis and associated bone resorption in physiological and pathological bone remodeling states (Figure 3).

### 4.2. PERK-eIF2α/PERK-NRF2

In the model of periodontitis induced by oral pseudomonas gingivalis in mice, the PERK-eIF2α signaling pathway has been shown to be involved in RANKL-induced osteoclast formation in BMMs [98]. Using RANKL-induced osteoclasts differentiated from BMMs and the ovariectomized mouse model, Jiachao Guo et al. showed that PERK positively regulated RANKL-induced osteoclast differentiation and bone resorption by activating NF-κB and MAPK signaling pathways and upregulated the expression of NFATc1, c-fos, and specific markers (TRAP, Cathepsin K, and MMP9). In addition, they showed that oxidative stress activated ER stress, which enhanced PERK phosphorylation and further promoted autophagy to induce osteoclastogenesis [107]. In addition, thapsigargin also activated the NF-κB pathway by increasing the expression of BiP and PERK, thereby promoting the expression of NAFA1, c-fos, and TRAP, and ultimately promoted osteoclastogenesis and bone resorption activity in the bone marrow cells of mice treated with RANKL and M-CSF [88]. In osteoclasts differentiated from BMMs in vitro, ER stress (induced by thapsigargin) positively regulated NFATc1 and c-fos through the BiP-PERK-eIF2α signaling pathway to promote osteoclast differentiation and bone resorption [87]. Osteolysis around the prosthesis leads to prosthesis loosening, often leading to failure after joint replacement. PERK-eIF2α played an important role in this process. Titanium particles not only promoted osteoclast formation through BiP-IRE1 but also mediated osteoclast formation and osteolysis through BiP-PERK-eIF2α-CHOP [91]. Guoxia Wang et al. reported that tricalcium phosphate granules activated the ER stress signaling pathway BiP-CHOP by stimulating the release of the inflammatory mediators TNF-α and IL-6, thereby promoting the expression of CTSK and TRAP and promoting osteoclast formation and osteolysis [108]. Boric acid had beneficial effects on bone remodeling. Mechanistic analysis showed that boric acid inhibited RANKL-induced activation of the PERK-eIF2α pathway, thereby suppressing the expression of the pro-osteoclastogenesis transcription factors c-Fos and NFATc1 in RANKL-treated RAW264.7 cells and a lipopolysaccharide (LPS)-induced bone loss model in mice [109].

The role of the PERK-eIF2α signaling pathway on osteoclastogenesis is controversial, depending on the animal and cell model used and the experimental conditions. Jie Li et al. used tail-suspended mice as a model of disuse osteoporosis and investigated the relationship between ER stress and disuse osteoporosis. Their results showed that the expression level of p-eIF2α was positively correlated with the count of osteoblasts and negatively correlated with the count of osteoclasts. Increased osteoclast numbers and reduced bone mass in unloaded mice were reversed by salubrinal, an inhibitor of dephosphorylation of eIF2α. Salubrinal increased p-eIF2α levels and inhibited the expression of RANKL, NFATc1, CTSK, and CHOP in unloaded mice. Moreover, salubrinal lessened the tunicamycin-induced increase in RAW264.7 cell viability, but not apoptosis [110]. Jie Li et al. also investigated the effect of salubrinal on osteoclast formation using ovariectomized osteoporotic mice as a model. Their results showed that salubrinal inhibited the expression of TRAP and CTSK by inhibiting the activity of Rac1 GTPase and NFATc1, thereby suppressing the migration, adhesion, differentiation, and bone resorption activity of osteoclasts [111]. Notably, salubrinal also inhibited the expression of CHOP and TNF-α in a mouse model of periodontitis, thereby suppressing the formation of TRAP-positive osteoclasts and alveolar bone resorption [112]. Kazunori Hamamura et al. found that salubrinal and guanabenz, as dephosphorylation inhibitors of eIF2α, both inhibited the RANKL-induced formation of TRAP-positive multinucleated osteoclasts and the expression of the TRAP. Furthermore, salubrinal inhibited osteoclast migration and adhesion. Mechanistic analysis revealed that salubrinal and guanabenz exerted their effects on osteoclastogenesis by inhibiting the activation of c-Fos and Jun-B in RAW264.7 cells and BMMs, followed by the inhibition of NFATc1 expression (which in turn inhibits c-Fos activation), but not by inhibiting NF-κB and MAPKs. Interestingly, they found that the decrease in NFATc1 protein expression was greater than the decrease in mRNA, which may be explained by the fact that salubrinal increased the level of p-eIF2α, resulting in the suppression of intracellular protein translation levels [100,113,114,115,116]. Long He et al. also found that salubrinal acted on the ER-stress-induced PERK-eIF2α pathway to reduce the expression of NFATc1 and inhibit osteoclastogenesis. In addition, they found that salubrinal also promoted ATF4 expression via phosphorylation of eIF2α to enhance osteoblast differentiation and RANKL expression, and ultimately contributed to osteoclastogenesis [117]. Therefore, salubrinal can act directly on osteoclasts to inhibit their formation or indirectly promote osteoclast formation by acting on osteoblasts to secrete RANKL.

The role of the PERK-NRF2 signaling pathway in osteoclastogenesis under ER stress has also been reported. Fluoride-involved stimulation of ER stress leads to different degrees of bone formation and bone resorption, in which PERK plays a pivotal role. Fluoride-involved bone resorption is associated with the promotion of RANKL expression by PERK, and NRF2 may be involved, but the exact mechanism has not been elucidated. Scholars suggested that NRF2 may play more of a role in the mechanism of bone formation [118]. In addition, Qinyi Liu et al. suggested that the possible mechanism of fluoride-induced osteoclast differentiation in OS732 cells was PERK-NRF2-mediated RANKL expression in the UPR signaling pathway, but interestingly, the addition of fluoride after siRNA PERK resulted in increased ATF4 and NRF2 expression, suggesting that fluoride did not only stimulate the activation of ATF4 and NRF2 through PERK signaling, which still needs further experiments to explore [119].

In conclusion, BiP-PERK-eIF2α had a positive effect on osteoclastogenesis (Figure 3). However, salubrinal, as a dephosphorylation inhibitor of eIF2α (which upregulated the expression of p-eIF2α), inhibited osteoclastogenesis mainly by suppressing the expression of NFATc1 (Figure 3). Notably, its inhibition of NFATc1 was due in part to the fact that phosphorylation of eIF2α led to a reduction in its activity, resulting in a decrease in mRNA translation in the general cells. Considering that p-eIF2α levels are upregulated in response to various stresses, including viral infection, radiation, and ER stress, and that PERK is only one of the four eIF2α protein kinases, salubrinal does not necessarily reflect the role of the UPR signaling pathway PERK-eIF2α in osteoclastogenesis [25,120]. In addition, salubrinal can also indirectly promote osteoclast differentiation by promoting the expression of RANKL in osteoblasts. The PERK-NRF2 signaling may mediate the increased expression of RANKL in fluoride-induced bone resorption, but a more precise mechanism has not been determined.

Therefore, the role of the PERK-eIF2α signaling pathway in osteoclastogenesis and a more detailed mechanism by which PERK-NRF2 regulates osteoclastogenesis need to be further explored and validated.

### 4.3. ATF6/CREBH

Following UPR initiation, S1P and S2P in Golgi co-cleave ATF6α from the ER. Zheng et al. showed that S1P promotes osteoclastogenesis and bone resorption activity, and the expressions of S1P and ATF6 were significantly reduced in S1P-deficient osteoclasts, but other ER stress-related proteins such as BiP, XBP-1, PERK, ATF4, EDEM1, DNAJB9, and CANX were not significantly changed, while ERN1 was only slightly reduced. Therefore, S1P may regulate osteoclast differentiation and bone resorption through other signaling mechanisms rather than through ER stress [121].

In response to intracellular ER stress, CREBH is activated by S1P and S2P cleavage (similar to ATF6). It has been found that ROS triggered ER stress and thus activated CREBH, which bound to the promoter of *NFATC1* and upregulated its expression, ultimately promoting RANKL-induced osteoclastogenesis [122]. In addition, thapsigargin increased the expression of BiP and ATF6 to promote the expression of NF-κB, which in turn promoted the expression of NFATc1, c-fos, and TRAP, and ultimately enhanced osteoclastogenesis and bone resorption activity in the bone marrow cells of mice treated with RANKL and M-CSF [88]. However, Eun-Gyeong Lee et al. found that ER stress positively regulated NFATc1 and c-fos to promote osteoclast differentiation and bone resorption activity through the BiP-PERK-p-eIF2α and BiP-IRE1α signaling pathways, without the involvement of ATF6 in a model using osteoclasts differentiated from BMMs [87].

Therefore, CREBH has a positive effect on osteoclastogenesis, and the regulation of ATF6 on osteoclasts is still controversial and needs further experiments to verify (Figure 3).

### 4.4. BIP

BiP is defined as a moonlighting protein (intracellular or extracellular protein with different functions) [123,124]. Although exogenous BiP has been found to inhibit osteoclast differentiation and bone resorption by suppressing CD115/c-Fms and RANK expression and downstream c-Fos and NFATc1 by attenuating NF-κB (classical and non-classical pathways) and MAPK signaling pathways, it will not be discussed here due to its lack of a clear link to ER stress [124]. As a stress protein, endogenous BiP is upregulated during ER stress, but its effect on osteoclasts has not been studied. Therefore, the relationship between BiP in UPR and osteoclastogenesis is still unknown.

### 4.5. UPR and the Expression of RANKL in Osteoblasts

The UPR increased the expression of RANKL in osteoblasts and osteocytes, thus indirectly promoting osteoclast formation and bone resorption [125]. The IRE1-XBP1 signaling pathway activated parathyroid hormone receptor-1 (PTH1R) transcription in osteoblasts, and PTH (parathyroid hormone)/PTH-related peptide promoted RANKL expression in osteoblasts via PTH1R, thereby promoting osteoclast differentiation [15]. Arl6ip5 (ADP-ribosylation-like factor 6-interacting protein 5) itself had no significant effect on osteoclastogenesis, but it affected RANKL expression in osteoblasts. *ARL6IP5* knockdown promoted RANKL expression in osteoblasts through ER stress and CHOP, thus indirectly promoting osteoclastogenesis [126]. In conclusion, UPR (PERK, CHOP, and IRE1-XBP1) promotes osteoblastogenesis indirectly by promoting RANKL secretion by osteoblasts.

## 5. Conclusions

With the aging of the world’s population, osteoporosis is having an increasing impact on health, causing a huge medical, economic, and family burden to patients and society. The over-differentiation of osteoclasts, as the primary bone-resorbing cells, plays a crucial role in the pathogenesis of osteoporosis. Exploring specific targets for the treatment of osteoclast over-differentiation remains a major research challenge. ER stress and its adaptive mechanism, UPR, occur in the context of the excessive aggregation of misfolded and unfolded proteins in the ER lumen and disturbed calcium ion homeostasis. It is worth noting that there are many common links between ER stress and osteoclasts, such as oxidative stress and an inflammatory reaction. The complex and close relationship between UPR and osteoclasts suggests an important role of UPR in osteoclast-related bone metabolism, and the UPR signaling pathway has been reported to play a crucial role in osteoclastogenesis in both physiological and pathological states. Here, we provided a comprehensive review of UPR, osteoclastogenesis-related signaling pathways, and the role of UPR in osteoclastogenesis and bone resorption. Available evidence suggested that the UPR signaling pathway (PERK, CHOP, and IRE1-XBP1) indirectly promoted osteoclast differentiation by promoting the secretion of RANKL from osteoblasts. The IRE1α signaling pathway promoted osteoclastogenesis by promoting the NF-κB, MAPK signaling, or the release of pro-inflammatory factors (IL-6, IL-1β, and TNFα). In addition, RANKL/RANK promoted osteoclastogenesis through the binding of XBP1 to the promoter of *NFATC1*. While PERK also promoted NF-κB, MAPK signaling promoted osteoclast differentiation. Furthermore, as a downstream signal of oxidative stress, PERK promoted autophagy to regulate osteoclast differentiation, but the specific mechanism has not been elaborated, which will be the future research direction. Interestingly, salubrinal (increasing the expression of p-eIF2α) acted directly on osteoclasts to inhibit osteoclastogenesis by inhibiting the expression of NFATc1, and indirectly promoted osteoclastogenesis by acting on osteoblasts (secreting RANKL). In addition, PERK-NRF2 promoted osteoclastogenesis by increasing the expression of RANKL, but the exact mechanism is also unclear. Thus, the specific role and mechanism of p-PERK and its downstream signaling in osteoclastogenesis are open to debate. In addition, CREBH was thought to increase NFATc1 expression to promote osteoclast differentiation, while the role of ATF6 in osteoclastogenesis is controversial. Finally, the role of BiP in osteoclastogenesis remains unaddressed. In summary, the IRE1α signaling pathway and CREBH signaling pathway can promote osteoclastogenesis, providing a new perspective for the treatment of diseases caused by osteoclast over-differentiation, such as osteoporosis. However, the roles and specific mechanisms of other signaling branches of UPR (such as p-PERK and ATF6) and BiP in osteoclastogenesis are still not well-explained, and further in vivo and in vitro experiments are required to elucidate them, which will be the focus of future studies.

## Figures and Tables

**Table 1:** Compounds related to ER stress and relevant mechanisms of action and other activities in this review.

Compound	Concentration	Role in ER Stress	Mechanism of Action	Other Activities	References
thapsigargin	0.5 nM	ER stress inducer	an inhibitor of the sarco/endoplasmic reticulum Ca^2+^-ATPase (SERCA) pump	initiation of ER stress and stress-induced apoptosis by inhibition of the SERCA pump; anti-tumor activity	[86,93]
tunicamycin	100 ng/mL	ER stress inducer	an inhibitor of protein glycosylation	induced apoptosis in colon or prostate cancer cells; inhibited the glycoprotein of coronavirus	[94,95]
4-Phenylbutyric acid	20 mM (in vivo) 1 and 2 mM (in vitro)	ER stress inhibitor	chemical chaperone (alleviating ER stress and preventing UPR dysfunction by enhancing protein folding capacity)	ammonia clearance; a histone deacetylase inhibitor	[96,97,98]
tauroursodeoxycholic acid	/	ER stress inhibitor	chemical chaperone	reduced oxidative stress; regulated and inhibited the apoptotic cascade response; protected mitochondria; reduced the inflammatory response	[99]
guanabenz	20 μM	inhibitor of dephosphorylation of eIF2α	interacting with protein phosphatase 1 (PP1)	treatment of hypertension (an agonist of α2 adrenergic receptors); antiparasitic (interfering with translation control)	[100,101]
salubrinal	1 and 2 mg/kg (in vivo) 1, 2, 5, 10, 20, and 30 μM (in vitro)	inhibitor of dephosphorylation of eIF2α	interacting with protein phosphatase 1 (PP1)	suppressed inflammation (by inhibiting dual-specificity phosphatase 2)	[100,102]

## Data Availability

The data presented in this study are available upon request from the corresponding author.
